# Recent advances in understanding and managing body dysmorphic disorder

**DOI:** 10.1136/eb-2017-102702

**Published:** 2017-07-20

**Authors:** Georgina Krebs, Lorena Fernández de la Cruz, David Mataix-Cols

**Affiliations:** 1 National and Specialist OCD, BDD and Related Disorders Clinic for Young People, South London and Maudsley NHS Foundation Trust, London, UK; 2 Social, Genetic and Developmental Psychiatry Centre, Institute of Psychiatry, Psychology and Neuroscience, King's College London, London, UK; 3 Centre for Psychiatry Research, Karolinska Institutet, Stockholm, Sweden; 4 Stockholm Health Care Services, Stockholm County Council, Stockholm, Sweden

**Keywords:** body dysmorphic disorder, diagnosis, treatment, cognitive behavioural therapy, antidepressants

## Abstract

Body dysmorphic disorder (BDD) is a relatively common and disabling psychiatric disorder characterised by excessive and persistent preoccupation with perceived defects or flaws in one's appearance, which are unnoticeable to others, and associated repetitive behaviours (eg, mirror checking). The disorder generally starts in adolescence, but often goes unnoticed and is severely underdiagnosed. Left untreated, BDD typically persists and causes marked functional impairment in multiple domains. This clinical review considers recent advances in the epidemiology and classification of BDD, including its reclassification in the fifth edition of the Diagnostic and Statistical Manual of Mental Disorders under the new ‘Obsessive–Compulsive and Related Disorders’ chapter. Key issues in assessment are outlined including the use of validated screening instruments to minimise misdiagnosis and the importance of risk assessment in this population given the high rates of suicidality and inappropriate use of cosmetic treatments. In addition, current knowledge regarding the causes and mechanisms underlying BDD are summarised. The recommended treatments for BDD are outlined, namely cognitive behavioural therapy (CBT) and antidepressants, such as selective serotonin reuptake inhibitors. Both CBT and pharmacotherapy have been shown to be efficacious treatments for BDD in adult populations, and evidence is emerging to support their use in young people. Although the majority of patients improve with existing evidence-based treatment, a large proportion are left with clinically significant residual symptoms. Priorities for future research are therefore discussed including the need to further refine and evaluate existing interventions with the goal of improving treatment outcomes and to increase their availability.

## Introduction

Body dysmorphic disorder (BDD) is characterised by excessive and persistent preoccupation with perceived defects or flaws in appearance. These perceived flaws are unobservable or appear only slight to others, but nevertheless give rise to significant distress and impairment in the sufferer.^1^ BDD sufferers can become preoccupied with any aspect of appearance, but the most common concerns relate to facial features, including nose, eyes, skin and hair.^2^ To meet diagnostic criteria for BDD, the appearance preoccupation cannot be better explained by concerns with body fat or weight in an individual who fulfils diagnostic criteria for an eating disorder.^1^ Diagnostic criteria for BDD also specify that at some point during the course of illness, the individual will have performed repetitive behaviours (eg, mirror checking, excessive grooming, skin picking, reassurance seeking) or mental acts (eg, comparing his or her appearance with that of others) in response to their appearance concerns.^1^

BDD typically follows a chronic course^3^ and is associated with marked functional impairment across multiple domains. Among adults, BDD results in high rates of occupational impairment, unemployment, social dysfunction and social isolation.^2^ Similarly, BDD in youth is associated with major functional impairment, including reduced academic performance, social withdrawal and dropping out of school.^2 4^ High comorbidity, for example with major depressive disorder, social anxiety disorder and obsessive–compulsive disorder (OCD), is frequently reported. BDD has also been associated with strikingly high rates of suicidality; reported rates of suicidal ideation range from 17%–77%, while rates of suicide attempts range from 3%–63%.^5^

Despite the seriousness of the disorder, BDD has received little empirical attention to date compared with related conditions, such as OCD. However, in recent years, increased efforts have focused on understanding the phenomenology, aetiology and treatment of the disorder. This article, aimed at non-specialist hospital doctors and general practitioners, as well as psychiatry and clinical psychology trainees, will review some key recent developments, with a focus on implications for clinical practice and avenues for future research. The current article is based on a comprehensive literature review. Relevant literature was identified using PubMed and PsycINFO up to April 2017.

## How is BDD classified?

A major advance in the field in recent years has been the reclassification of BDD in the diagnostic manuals as well as the refinement of its diagnostic criteria. In the revised version of the fourth edition of the Diagnostic and Statistical Manual of Mental Disorders (DSM-IV),^6^ BDD was listed as a diagnosis within the somatoform disorders section,^6^ but several significant changes were made in DSM-5.^1^ First, in light of the phenomenological overlap and high rates of comorbidity between BDD and OCD,^7^ BDD was classified under the new Obsessive–Compulsive and Related Disorders chapter along with OCD, hoarding disorder, trichotillomania (hair-pulling disorder) and excoriation (skin picking) disorder. Second, a new diagnostic criterion was included, specifying repetitive behaviours or mental acts as a key feature of the disorder. This criterion increased the specificity of the diagnosis, potentially helping to differentiate BDD from other disorders such as social anxiety disorder and depression. Third, two specifiers were included to identify meaningful BDD subgroups. The insight specifier enables clinicians to identify patients with delusional dysmorphic beliefs without assigning a separate diagnosis of delusional disorder, which could lead to inappropriate treatment with antipsychotic medication.^8^ The muscle dysmorphia specifier describes BDD patients who are preoccupied with the idea that their body build is too small or insufficiently muscular. This specifier has potential clinical utility since muscle dysmorphia, which is more common in males, has been found to be associated with poorer quality of life, higher rates of suicide and higher rates of substance abuse, including anabolic steroid abuse.^9^

In the current edition of the International Classification of Diseases (ICD-10),^10^ BDD is not an independent diagnostic category, but instead is listed as an inclusion term under hypochondriacal disorder. Notably, BDD symptoms are also referred to under several other diagnostic categories (eg, delusional disorder, schizotypal disorder and other persistent delusional disorder), potentially giving rise to diagnostic confusion and inappropriate treatment.^8^ Although the publication of the new ICD-11 is not planned until 2018, current proposed recommendations concerning the classification of BDD closely mirror the DSM-5 criteria, including listing BDD as a separate diagnosis within a new Obsessive–Compulsive and Related Disorders chapter.^11^

## How common is BDD?

In a recent systematic review, the weighted prevalence of BDD was estimated to be 1.9% in community samples of adults and 5.8%–7.4% in psychiatric settings, highlighting the importance of clinical vigilance for the disorder.^12^ Comparable rates have been found for adolescents, with prevalence estimates ranging from 1.7%–2.2%^12 13^ in the community and 6.7%–14.3% in psychiatric inpatient settings.^12^ BDD has been shown to be more common in older adolescents,^13^ consistent with reports that the mean age of onset is around 16 years.^14^ No study to date has examined the prevalence of BDD in young people under the age of 12 years, thus it remains unclear how common BDD is in childhood. Furthermore, the majority of community-based prevalence studies have been conducted within Europe and North America, and it is therefore unknown whether rates vary across different cultures.^12^ Such knowledge would assist in identifying at risk populations, enabling focused efforts to promote diagnosis and treatment.

With respect to sex differences in prevalence, findings have been inconsistent, with some studies suggesting that BDD is more common in females^12^ and others indicating equivalent prevalence rates in both genders.^13^ These discrepancies may reflect methodological differences across studies including variation in the study setting, with higher female to male ratios typically found in community compared with clinical settings.^12^ In this vein, there is some suggestion that subclinical BDD symptoms are more common in females but that the prevalence of diagnosable BDD is equivalent in both sexes.^13^ The features of BDD are broadly similar in males and females, but evidence suggests that males are more likely to be preoccupied with their genitals and thinning hair, while females are more likely to be preoccupied with hips, breasts, legs and excessive body hair.^9^ Thus, clinicians should be aware of potential differences in the clinical presentation of BDD in males and females.

## How should BDD be assessed?

Despite its prevalence and impact, current evidence suggests that BDD often goes undiagnosed.^12^ This may partly reflect reluctance of BDD patients to seek mental health support due to shame and embarrassment about symptoms, poor insight and a desire for non-mental health treatment such as cosmetic surgery.^15^ However, even when sufferers do present to mental health services, they are unlikely to spontaneously disclose their appearance concerns.^12^ Thus, BDD symptoms often need to be explicitly asked about during the anamnestic interview. Lack of spontaneous symptom disclosure combined with limited awareness of BDD among clinicians may result in misdiagnosis, with BDD symptoms being misclassified into other disorders that are common comorbidities, such as depression and social anxiety disorder^12^ (see table 1 for information on differential diagnosis). Furthermore, among adolescents in particular, there may be difficulty differentiating mild BDD symptoms from normative appearance concerns.^4^

**Table 1:** Common differential diagnoses in BDD

	Similarity with BDD	Key differentiating features
OCD	Time consuming, repetitive behaviours which can include grooming rituals	Unlike in BDD, grooming rituals in OCD are not driven by an attempt to correct perceived appearance flaws. They may instead be driven by contamination fears or ‘just right’ urges.
Excoriation disorder	Repetitive skin picking	Skin picking in excoriation disorder is not driven by an attempt to improve appearance, whereas skin picking in BDD is intended to improve the appearance of perceived defects in the skin.
Trichotillomania	Repetitive hair pulling	Hair pulling in trichotillomania is not driven by an attempt to improve appearance, whereas hair pulling in BDD is intended to improve the appearance of perceived defects in facial or body hair.
Eating disorders	Distressing and impairing preoccupation with appearance	Appearance preoccupation in eating disorders is focused on body weight and shape, leading to dysfunctional eating behaviours in an attempt to lose weight.
Social anxiety disorder	Avoidance of and distress in social situations	Social avoidance in social anxiety disorder is driven by a fear of saying or doing something to embarrass oneself. In BDD, social anxiety is exclusively linked to a fear of negative judgements about perceived appearance defects.
Depression	Can involve feelings of ugliness as part of pervasive low self-esteem	Concerns about appearance are not the primary preoccupation in depression and not typically associated with the repetitive behaviours that are characteristic of BDD (eg, mirror checking, grooming).

BDD, body dysmorphic disorder; OCD, obsessive–compulsive disorder.

**Figure 1:**
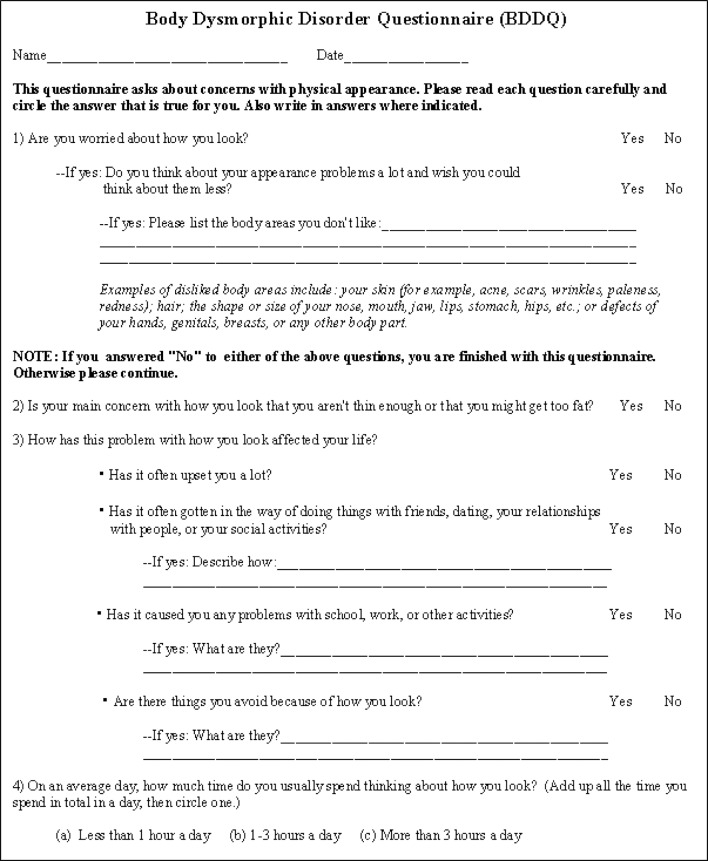
Screening measure for body dysmorphic disorder: the Body Dysmorphic Disorder Questionnaire ©THE BROKEN MIRROR: UNDERSTANDING AND TREATING BODY DYSMORPHIC DISORDER by Katherine A. Phillips (2005): Questionaire: BDDQ for Adolescents (Appendix C, p.380). "By Permission of Oxford University Press, USA".

Despite these diagnostic challenges, accurate diagnosis of BDD in primary and secondary care settings can be greatly aided by use of brief screening instruments. For example, the Body Dysmorphic Disorder Questionnaire (see figure 1) is a four-item measure that has high sensitivity (94%–100%) and specificity (89%–93%) in detecting BDD in a range of settings.^16 17^ On the other hand, the BDD version of the Yale-Brown Obsessive–Compulsive Scale (BDD-YBOCS) is a widely used 12-item semi-structured clinician-administered interview that rates the severity of BDD symptoms during the past week.^18^ Due to the fact that this instrument is lengthy to administer and requires some specialist training, it may not be feasible to use many clinical settings, but it is the gold-standard measure to assess the severity of BDD symptoms and it is generally the main outcome measure used in clinical trials. The BDD-YBOCS has good psychometric properties and is sensitive to changes in BDD severity.^18^ As a convention in the field, a pre-to-post-treatment reduction of ≥30% on the BDD-YBOCS score denotes ‘treatment response’.^19^

Assessment of BDD should always include consideration of risk. In a recent meta-analysis, patients with BDD were found to be more than twice as likely to have attempted suicide compared with controls (mainly psychiatric patients with diagnoses including eating disorders and OCD), highlighting the importance of assessing suicidality in this population.^5^ In addition, there is accumulating evidence that BDD sufferers commonly seek cosmetic treatments in an attempt to correct their perceived appearance defect, with 33%–76% of patients undergoing surgical and/or minimally invasive cosmetic interventions.^20^ This is concerning given that cosmetic treatment in BDD is typically associated with negative outcomes, including poor patient-reported satisfaction, persistence of BDD symptoms, worsening of BDD symptoms and higher levels of postoperative complications.^20^ For this reason, clinical assessment of BDD should also routinely include screening regarding desires and plans for cosmetic treatments, and patients should be encouraged towards evidence-based treatment for BDD as opposed to cosmetic interventions.

## What causes BDD?

Diathesis stress models of BDD propose that the disorder results from an interplay between biological predisposing factors and environmental stressors. Results of twin studies indicate that genetic factors account for approximately 42%–44% of the variance in BDD-like symptoms, with the remaining variance being account for by non-shared environmental influences.^21 22^ Genome-wide association studies have yet to be conducted in BDD, and thus no specific risk genes have been identified to date. The specific aspects of the environment that contribute to the development of BDD also remain unknown. Research on environmental risk factors in BDD is sparse and most studies have serious methodological limitations, including an over-reliance on cross-sectional and retrospective designs, lack of multiple-informant assessment methods and inadequate control of potential confounding variables such as comorbidity and genetic factors. Nevertheless, a range of environmental factors have been suggested to influence the development of BDD, including childhood abuse, peer teasing and peer victimisation. Studies have shown that adults with BDD report high levels of childhood maltreatment, with up to 79% of patients reporting abuse.^23^ Furthermore, retrospectively reported rates of abuse are elevated in patients with BDD compared with healthy controls^24^ and patients with OCD,^25^ although the cross-sectional nature of these studies prevents any interference regarding causality. Bullying has also been shown to be associated with BDD.^26^ Several recent studies have shown associations between self-reported appearance-related teasing and BDD symptoms in analogue samples^27 28^ and clinical samples,^29^ particularly when the teasing is by members of the opposite sex.^27^ In one of the only longitudinal studies of environmental risk factors in BDD, peer victimisation in school students (as reported by the peer group) was prospectively associated with the development of BDD symptoms 12 months later,^30^ in line with suggestions that experiences of bullying may play a causal role in BDD. Although further research is clearly needed, understanding the role of environmental risk factors could have important implications for the prevention and early intervention in BDD.

## What evidence-based treatments are available for BDD?

In line with the extant evidence base, clinical guidelines recommend cognitive behavioural therapy (CBT) and serotonin reuptake inhibitors (SRIs) in the treatment of BDD.^31^ In clinical trials, CBT for BDD typically involves 12–22 weekly sessions,^32 33^ with a key therapeutic strategy being exposure with response prevention (E/RP). E/RP involves the gradually confronting of feared situations (eg, bright lights, mirrors, social situations) and resisting the urge to perform safety-seeking behaviours (eg, camouflaging, applying excessive make-up, focusing attention internally) to neutralise distress, with the goal of achieving anxiety habituation. Additional strategies that have been used in CBT for BDD include psychoeducation, motivational enhancement techniques, cognitive restructuring, mirror retraining and attention training.^4 32 33^

In adult populations, six randomised controlled trials (RCTs) have demonstrated CBT to be efficacious in reducing BDD severity compared with no treatment or waitlist control conditions,^33–36^ supportive therapy^37^ and anxiety management.^32^ Furthermore, the first RCT of CBT for BDD in adolescents was recently published, showing developmentally tailored CBT to be efficacious compared with a control condition.^4^ A meta-analysis of the seven RCTs conducted to date concluded that CBT as an efficacious treatment for BDD symptoms (effect size (ES)=1.22) and associated features, such as depression (ES=0.49) and insight/delusionality (ES=0.56).^38^ While these outcomes are encouraging, 46%–60% of BDD trial participants do not respond sufficiently to CBT, and remission rates are low.^38^ Outcomes may be even less favourable in routine clinical practice, where patients are unselected and clinicians may be less experienced in the treatment of BDD. There is a clear need to evaluate the long-term effects of CBT for BDD since few studies have addressed this question to date.^39–41^ A recent 12-month follow-up study of adolescents who had received CBT for BDD indicated that overall gains are maintained but that a significant proportion continue to experience clinically significant symptoms and remain vulnerable to a range of potential risks and negative outcomes (eg, cosmetic surgery, suicidal behaviour, risky sexual behaviours).^39^ For this reason, longer term monitoring of patients with BDD following CBT is recommended.

Although CBT is an efficacious treatment for BDD, many patients continue to experience significant symptoms and there is a pressing need to improve existing CBT packages for BDD to enhance outcomes. Such efforts can be informed by better understanding the mechanism underlying the development and maintenance of BDD and its recovery. In parallel, empirical attention should also be given to developing evidence-based methods for disseminating CBT for BDD, given that the treatment is not widely available. For example, therapist-guided internet-based CBT has the potential to greater increase availability and access. A recent RCT found that 56% of patients with BDD responded to a 12-week internet-based CBT package with just 13 min of therapist support per week on average.^37^ Further trials comparing the efficacy or non-inferiority of low-intensity remote interventions like internet-based BDD^37^ for mild to moderately severe (non-suicidal) BDD against gold standard face-to-face CBT are needed. Such low intensity interventions could represent a first treatment option in a stepped care model, which could potentially increase availability of CBT and optimise the limited available resources.

A range of SRIs have been used in the treatment of BDD, including fluoxetine,^42^ fluvoxamine,^43^ citalopram,^44^ escitalopram^45^ and clomipramine.^46^ Most evidence for the efficacy of pharmacotherapies in BDD comes from open trials, and only four RCTs of pharmacotherapy have been conducted to date,^42 45–47^ which have found response rates ranging from 53%–70%.^42 46^ The most recent RCT conducted was a two-phase trial.^45^ Phase 1 was a 14-week open trial in which patients with BDD were treated with escitalopram. In phase 2, treatment responders were randomised to escitalopram continuation or placebo for a further 6 months, in a double-blind design. Results showed that 40% of placebo group relapsed compared with only 18% of the escitalopram-continuation group, and overall the escitalopram-continuation group made further gains. The key implications of this study are that patients with BDD should remain on SRIs medication for relatively long periods to reduce the likelihood of relapse occurring. While dose-finding studies have not been conducted in BDD, available data and clinical experience indicate that BDD often requires SRI doses that are higher than those required to treat depression and similar to those required to treat OCD.^48^ Of note, clinical experts in the field have suggested that doses required to treat BDD often exceed the regulatory limit.^48^ In addition, tailoring SRI titration is recommended, based on factors such as severity of illness, risk, how well the medication is tolerated and patient preference.^49^

Further research is needed to establish the relative efficacy of different SRIs and to compare pharmacotherapy to CBT in RCTs and meta-analytic studies. There is also a need to further evaluate potential augmentation strategies for BDD patients who do not respond to SRIs. To date, research on augmentation strategies in BDD is limited to one small open trial and one RCT which evaluated pimozide and olanzapine augmentation of fluoxetine, respectively.^47 50^ These studies did not find beneficial effects of augmentation, but this warrants investigation as clinical experience and guidelines suggest that SRI augmentation with an atypical antipsychotic can be beneficial.^31^

## Conclusions

In summary, BDD is a relatively common and potentially debilitating disorder, but research on BDD is still its infancy compared with other psychiatric disorders. There is a pressing need to increase awareness of this serious condition and to promote detection, diagnosis and treatment. Current research and clinical guidelines indicate that CBT and SRI medication are the treatments of choice for BDD. Expert clinical experience suggests that longer courses of CBT (ie, more sessions) and higher doses of SRI medication are often required to treat BDD compared with other common psychiatric disorders such as depression. Severe BDD cases may be best managed in specialist settings given the high levels of morbidity, risk and complexity of treatment. While many patients respond well to existing evidence-based treatment, a significant proportion experience enduring symptoms. Ongoing research into the aetiology of BDD and factors predicting treatment response may shed light on the mechanisms underlying the development and maintenance of the disorder, ultimately leading to new and improved treatment possibilities.
